# Discovery and biological evaluation of novel CARM1/HDAC2 dual-targeting inhibitors with anti-prostate cancer agents

**DOI:** 10.1080/14756366.2023.2241118

**Published:** 2023-08-01

**Authors:** Sudong Liang, Yifei Geng, Miao-Miao Niu, Yan Zhang, Weiping He, Jindong Li, Li Yang, Zhen Xu

**Affiliations:** a Department of Urology, Pharmacy and Oncology, The Affiliated Taizhou People’s Hospital of Nanjing Medical University, Taizhou, China; b Department of Pharmaceutical Analysis, China Pharmaceutical University, Nanjing, China

**Keywords:** Prostate cancer, CARM1, HDAC2, dual-targeting inhibitors, structure-based virtual screening

## Abstract

Prostate cancer (PCa) is a clinically heterogeneous disease with a progressively increasing incidence. Concurrent inhibition of coactivator-associated arginine methyltransferase 1 (CARM1) and histone deacetylase 2 (HDAC2) could potentially be a novel strategy against PCa. Herein, we identified seven compounds simultaneously targeting CARM1 and HDAC2 through structure-based virtual screening. These compounds possessed potent inhibitory activities at the nanomolar level *in vitro*. Among them, CH-1 was the most active inhibitor which exhibited excellent and balanced inhibitory effects against both CARM1 (IC_50_ = 3.71 ± 0.11 nM) and HDAC2 (IC_50_ = 4.07 ± 0.25 nM). MD simulations presented that CH-1 could stably bind the active pockets of CARM1 and HDAC2. Notably, CH-1 exhibited strong anti-proliferative activity against multiple prostate-related tumour cells (IC_50_ < 1 µM). *In vivo,* assessment indicated that CH-1 significantly inhibited tumour growth in a DU145 xenograft model. Collectively, CH-1 could be a promising drug candidate for PCa treatment.

## Introduction

As the most common male urological malignancy globally, prostate cancer (PCa) is one of the leading causes of cancer-related mortality in men with poor prognosis^1^^,^^2^. Owing to the insidious onset of PCa, most patients have obvious symptoms in the middle and late stages^3^. Standards of care for advanced prostate cancer includes endocrine therapy, chemotherapy, and targeted therapy^4^. Although these therapies are initially effective, they lead to the recurrence of the disease and rapid growth of cancer cells, culminating in castration-resistant prostate cancer (CRPCA), which is highly aggressive and prone to bone metastasis^4^. Currently, there were an estimated 10 million new confirmed cases of PCa worldwide, and more than 400,000 deaths annually^5^^,^^6^. Although there are numerous FDA-approved agents for prostate cancer treatment, they are severely limited by the resistance to androgen deprivation therapy (ADT) and paclitaxel chemotherapy drugs^7^. Thus, there is an urgent need to discover novel and efficient drugs for PCa treatment. With the emergence of novel targeted therapeutics and biomarkers in cancer, mounting evidence suggests that except genomic alterations, alterations in epigenetic modifications such as histone methylation and acetylation are prevalently associated with the progression of PCa^8^^,^^9^.

**Figure 1.**
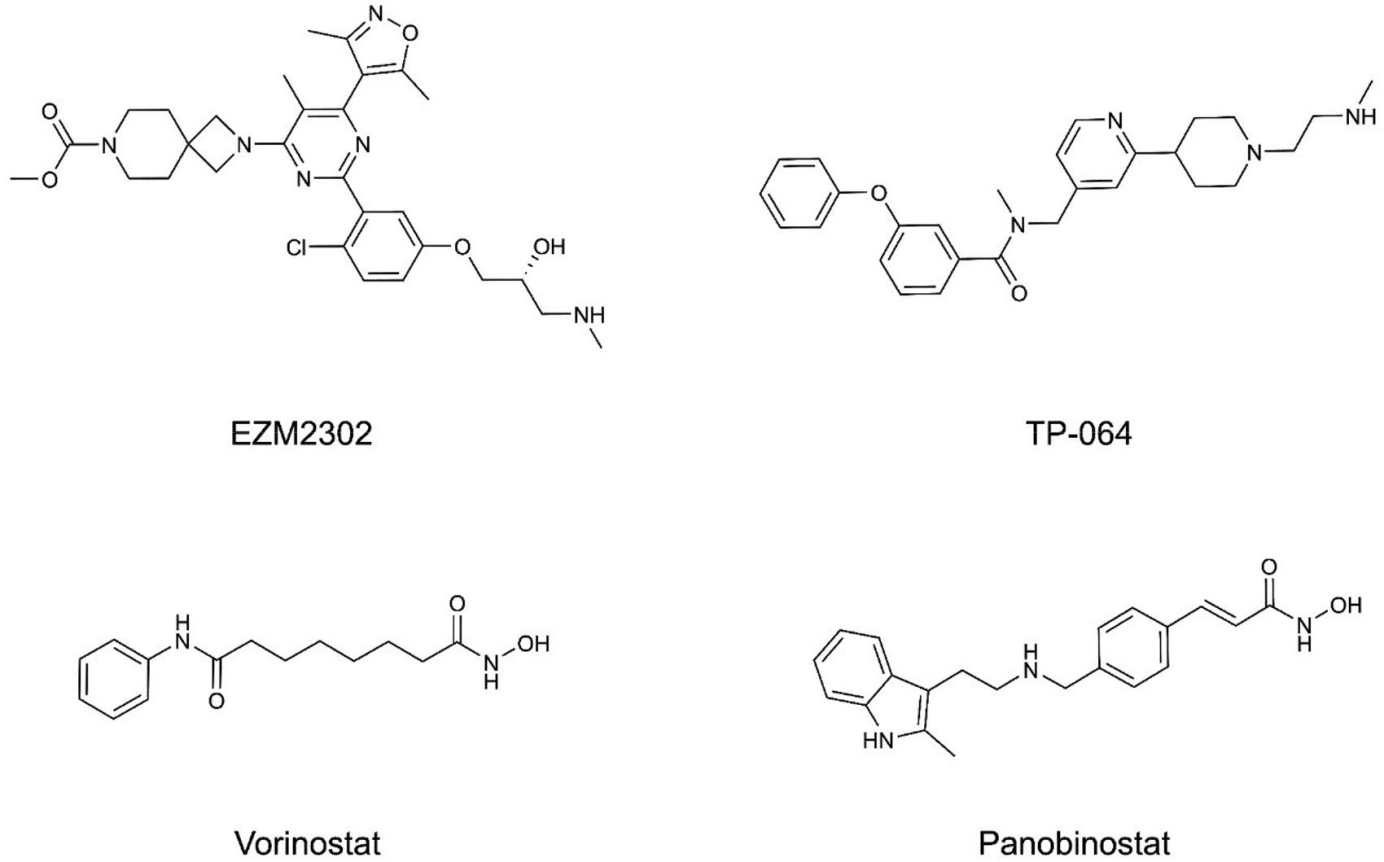
Reported CARM1 and HDAC inhibitors.

Coactivator-associated arginine methyltransferase 1 (CARM1), also referred to as PRMT4 is a member of the protein arginine methyltransferases (PRMTs) family^10^. PRMTs transfer a methyl group from S-adenosyl-L-methionine (SAM) to the side chain of specific arginine residues of the substrate protein^11^. According to the location of methylation, PRMTs are classified into three categories: type I, II, and III^12^. CARM1 is one of the type I PRMTs and converts arginine into monomethyl arginine (MMA) and asymmetric dimethylarginine (ADMA)^10^^,^^13^. CARM1 can modify the methylation of histone H3R17 and H3R26, as well as other chromatin-associated nonhistone proteins, like chromatin remodelling factor BAF155, and RNA-binding protein HuR^14–16^. Therefore, CARM1 is frequently involved in diverse biological processes, such as DNA repair, transcriptional coactivation, mRNA splicing, and autophagy^17–20^. The aberrant expression of CARM1 is related to PCa^21^^,^^22^. CARM1 is essential in the oncogenic growth of prostate cancer, and overexpression of CARM1 in PCa correlates with androgen signalling, cell cycle, and EMT regulators^21^. Importantly, dysregulation of CARM1 is also implicate in other tumours like breast cancer, pancreatic cancer, and non-small cell lung cancer (NSCLC)^23–25^. In the past few years, several CARM1 inhibitors have been reported, most of which are small molecule inhibitors containing the alanine amide moiety and ethylenediamine side chain, but they showed low cellular activity and poor selectivity against other type I PRMTs^26–28^. Since then, great efforts have been made to find inhibitors with high *in vivo* activity, such as EZM2302 and TP-064 (Figure 1), which were discovered with potent and selective *in vivo* activities in human multiple myeloma models^29–31^. However, none of the CARM1 inhibitors have been approved yet.

Histone deacetylases (HDACs) are essential enzymes in histone modifications, which catalyse and regulate the deacetylation of lysine residues in histones^32^. Histone deacetylation is a crucial epigenetic modification mediating conformational changes in nucleosomes, affecting chromatin structure and gene expression^33^. There are 18 HDAC enzymes in humans, and their functions are frequently perturbed in cancer^32^^,^^34^. Specifically, HDAC2 is overexpressed, mutated, or abnormally recruited in cancer cells^35^. Indeed, the oncogenic role of HDAC2 has been confirmed in prostate cancer^36^. Previous evidence has suggested that HDAC2 is necessary for both beta-adrenergic signalling and CREB activation to promote prostate cancer cells proliferation^37^. Consequently, inhibition of HDAC2 is regarded as a rational therapeutic strategy to reverse cancer-involved epigenetic changes^38^^,^^39^. In recent years, numerous HDAC inhibitors with various chemical structures, including hydroxamic acids, benzamides, cyclic peptides, and carboxylic acids, are currently in clinical trials^40^. Several clinically successful HDAC inhibitors have received FDA approval, such as vorinostat (SAHA), panobinostat^41^^,^^42^. Most clinical HDAC drugs as monotherapy demonstrated positive results in haematological malignancies, but unfortunately, their clinical findings in solid tumours are not satisfactory^43–45^. Overall, the exploration of HDAC inhibitors for treating solid tumours remains enormous challenges.

Accumulating evidence has confirmed that prostate cancer can easily acquire resistance to any single protein or kinase inhibitor, and thus currently single-targeted agents are frequently limited by drug resistance^46^^,^^47^. In addition, single HDAC inhibitors are poor in the treatment of solid tumours^43^. An alternative method to circumvent these drawbacks is to explore novel CARM1 and HDAC2 dual-targeting inhibitors. Recently studies have reported that CARM inhibitors displayed excellent *in vivo* efficacy for solid tumours^48^. Thus, concurrent inhibition of CARM1 and HDAC2 may enhance the sensitivity to solid tumour cells, and exert synergistic therapeutic benefits over single drugs. Furthermore, dual inhibitors can circumvent drug-drug interactions compared to combination treatment to a certain extent^49^. Encouragingly, many successful cases of dual inhibitors based on HDAC have been reported, such as dual STAT3/HDAC inhibitors and dual FGFR/HDAC inhibitors^50^^,^^51^. All of these demonstrated significant anticancer efficacy in treating malignancies. Based on these findings, CARM1 and HDAC2 dual inhibition is considered as a promising therapeutic approach. To date, none of the dual CARM1/HDAC2 inhibitors have been reported.

Nowadays, structure-based virtual screening is a valid tool for the identification and optimisation of lead compounds^52^^,^^53^. With the benefits of low cost and less time consumption, it is rapidly gaining acceptance as a popular alternative for high-throughput screening (HTS)^54^. Herein, we successfully identified seven novel and highly potent CARM1/HDAC2 dual inhibitors through structure-based virtual screening based on pharmacophore screening and molecular docking. These compounds showed extremely excellent inhibitory activities against human prostate cancer cells (IC_50_ < 1 µM) *in vitro.* Among them, CH-1 is the most potential inhibitor which significantly inhibited the tumour growth *in vivo.* These results indicated that CH-1 is a promising candidate for further exploration.

## Materials and methods

### Materials

The American Type Culture Collection (ATCC) (Manassas, VA, USA) provided the human cancer cell lines including DU145, RM-1, LAPC4, 22RV1, and PC-3, which was then cultured in Dulbecco’s modified Eagle’s medium (DMEM) with 1% penicillin-streptomycin and 10% foetal bovine serum (FBS) in a humidified environment with 5% CO2 at 37 °C. Recombinant human CARM1 and HDAC2 proteins were purchased from Abcam (Cambridge, MA, USA). Hit compounds were purchased from WuXi AppTec.

### Pharmacophore-based screening

As the previously reported method described, the following screening was conducted using the Molecular Operating Environment (MOE, Chemical Computing Group Inc, Montreal, Quebec, Canada) program^55^. The X-ray crystallographic structure of CARM1 (PDB ID: 6ARJ) was obtained from the Protein Data Bank (PDB) and pre-processed by the MOE using Amber14: EHT force field including removing water, adding hydrogen atoms, calculating gasteiger partial charges, and performing energy minimisation. A small molecule database containing 35 000 compounds from the CPU laboratory was used for virtual screening. All the compounds were further converted into a three-dimensional structure through the energy optimisation algorithm of MOE. Subsequently, the Surfaces and Maps tool was used to analyse the structure of CARM1. The ligand interaction tool was applied to analyse the interaction between compounds and proteins. Based on crucial interactions between CARM1 and ligand. The representative features of the CARM1 active site were manually determined using the pharmacophore query editor. Each pharmacophore feature represented the crucial interaction points with the key residues on ligand binding of the CARM1. The MOE was then used to search the small molecule database for potential hits. RMSD values between the pharmacophore query features and matching ligand annotation points were used to measure the matching degree of hit compounds.

### Validation of pharmacophore model

As the previously published method described, the selectivity of the generated CARM1 pharmacophore model was validated using the Güner–Henry (GH) scoring method^55^. A testing set database including 1484 inactive molecules and 16 active molecules was constructed. The inactive compounds were downloaded from the enhanced Database of Useful Decoys (DUDe) and the active compounds were collected from the reported literature^26^^,^^56^. The validated model was employed as a 3D query to filter the testing database applying the pharmacophore search function of MOE. The hit compounds were analysed based on the following formula:

$$GH=(\frac{Ha(3A+Ht)}{4HtA})(1-\frac{\left(Ht-Ha\right)}{\left(D-A\right)})$$

The parameters containing total molecules in the database (D), the total number of actives in the database (A), total hits (Ht), active hits (Ha), % yield of actives, % ratio of actives, enrichment factor (E), false negative, false positives, and goodness of hit score (GH) were calculated as a statistical metric. The GH score ranges from 0 (representing a null model) to 1 (representing an ideal model).

### Molecular docking

The docking screening was performed as previously reported^55^. We retrieved the X-ray crystallographic structure HDAC2 (PDB ID: 4LXZ) from the PBD. The protein was imported into the MOE program and prepared by removing water, adding hydrogen atoms, calculating partial charges, and performing energy minimisation in Amber14: EHT force field. The Surfaces and Maps tool is used to analyse the surfaces of protein. After screening based on the generated pharmacophore model, the Dock protocol was used for molecular docking. The compounds selected by the pharmacophore screening were further docked to the above-prepared CARM1 and HDAC2 proteins, respectively. The docking scoring function served to calculate the binding free energy. Subsequently, we set reasonable cut-off values separately for CARM1 docking binding energy and HDAC2 docking binding energy as a filtering criterion. Eventually, the hit compounds that simultaneously comply with the CARM1 and HDAC2 docking scoring thresholds were selected.

### Molecular dynamics simulation

According to the method reported previously, the molecular dynamics simulations were implemented by GROMACS 2021.5 in the AMBER99SB-ILDN force field with periodic boundary conditions^57^. As the first step, the complex was dissolved by TIP3P water molecules in a cube box at a distance of 1.0 nm from the complex, and Na^+^ and Cl^−^were added in order to neutralise the system. After that, the energy minimisation of the system was performed. We employed the steepest descent algorithm and set 5000 steps in this process. In addition, a 1 ns NVT simulation was used to maintain the system temperature at 300K with a V-rescale thermostat. A further NPT simulation for 1 ns was conducted by Parinello–Rahman barostat to keep the constant system pressure (1 bar). Lastly, 50 ns MD simulations were performed on the system, and the trajectory data were recorded at 10 ps time intervals.

### In vitro CARM1 inhibition assay

*In vitro* CARM1 inhibition assays were conducted as previously described^29^. Before the reaction began, CARM1 was preincubated with compounds for 30 min at room temperature. Then, 0.25 nM CARM1, 30 nM ^3^H-S-adenosyl-methionine (SAM), and 250 nM biotinylated peptide were added in a buffer including 20 mM bicine, 1 mM tris (2-carboxyethyl) phosphine, 0.005% bovine skin gelatine and 0.002% Tween-20. Assay was performed at the condition of pH 7.5. The assay was quenched through the addition of 300 µM unlabelled SAM. The quantity of ^3^H-labeled peptide produced was measured by Flashplate on a Topcount reader.

### In vitro HDAC2 inhibition assay

*In vitro* HDAC2 inhibition assays were performed as previously stated^58^. Briefly, 50 µL of various concentrations of tested compounds were mixed with 10 µL of HDAC2 enzyme solution. After 5 min of 37 °C incubation, 40 µL of the luorogenic substrate Boc-Lys(acetyl)-AMC was added. The mixture was incubated at 37 °C for 30 min and stopped by adding 100 µL of developer containing trypsin and TSA. Then, fluorescence intensity was determined 20 min later at excitation and emission wavelengths of 390 and 460 nm, respectively, using a microplate reader. The IC_50_ values were determined by the GraphPad Prism 6 program based on a sigmoidal dose − response equation, and the inhibition rates were calculated using the fluorescence intensity readings of the tested wells in comparison to those of the control wells.

### MTT cell growth assay

Based on the experimental method published previously, cell lines were prepared in cell suspension (5 × 10^4^ cells/mL) and seeded into a 96-well plate^59^. After overnight cultivation, the cells were exposed to CH-1 at various concentrations and incubated at 37 °C for 72 h. Then MTT solution (0.5 mg/ml) was added into each well and continued to cultivate for 4 h. Using dimethyl sulfoxide (DMSO) to dissolve insoluble crystals, the cell viability was determined based on the absorbance measured by a microplate reader at the wavelength of 570 nm.

### In vivo anticancer activity

The method of *in vivo* assay was described previously^60^. We bought healthy female BALB/c nude mice from the animal experiment centre of Yangzhou University. All experiments involving animals were approved by the Animal Ethics Committee of China Pharmaceutical University. Mice were subcutaneously injected on the right flank with DU145 cells (200 µl, 1 × 10^7^ cells). When tumour sizes reached 80–100 mm^3^, mice were divided into four groups at random. Every four days for a total of six times, each group was injected intraperitoneally with the vehicle, CH-1 (5 mg/kg), CH-1 (10 mg/kg), and CH-1 (20 mg/kg). Tumour volume was measured every four days and calculated by the formula (c × c × d)/2 (c, the smallest diameter; d, the largest diameter).

## Results

### Virtual screening of dual CARM1/HDAC2-targeting inhibitors

**Figure 2.**
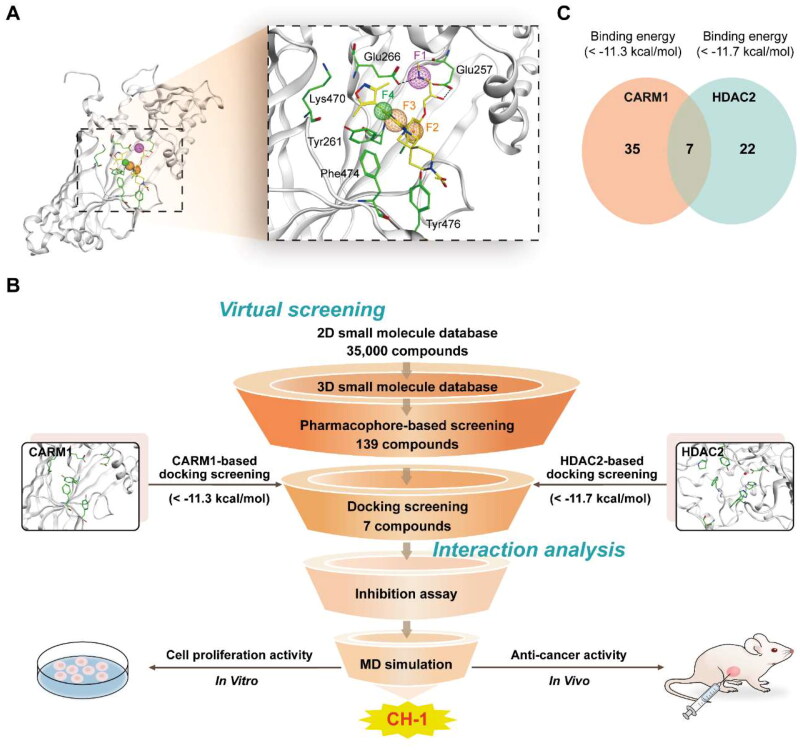
Identification of compounds targeting CARM1/HDAC2 through the integrated screening strategy. (A) Details of the CARM1-based pharmacophore model (F1: H-bond donor feature, F2 and F3: aromatic centre features, F4: hydrophobic centroid feature). Pharmacophore features of CARM1 were represented as spheres. Hydrogen bonds were indicated by dashed black dotted lines. (B) The workflow of combinatorial virtual screening process of dual CARM1/HDAC2-targeting inhibitors. (C) Identification of 7 intersection compounds with lower CARM1-binding energies (< −11.3 kcal/mol) and HDAC2-binding energies (< −11.7 kcal/mol).

In order to identify dual CARM1/HDAC2 inhibitors, we first constructed pharmacophore models of CARM1 based on the CARM1 crystal structure (PDB ID: 6ARJ). Ligand-protein interactions were analysed using the ligand interaction tool. As shown in Figure 2(A), we selected to generate four representative pharmacophore features (F1-F4), consisting of one hydrogen-bond donor feature (F1), two aromatic centre features (F2 and F3), and one hydrophobic centroid feature (F4). Feature F1 corresponded to the critical residues Glu266 and Glu257, which formed hydrogen bonding interactions with the ligand. The aromatic features F2 and F3 corresponded to residues Tyr476, Tyr261, and Lys470. Feature F4 corresponded to residue Phe474, Tyr261, and Tyr476, forming hydrophobic interactions. The final CARM1 pharmacophore model was generated based on the four pharmacophore features and then prepared for the next step of docking screening.

**Figure 3.**
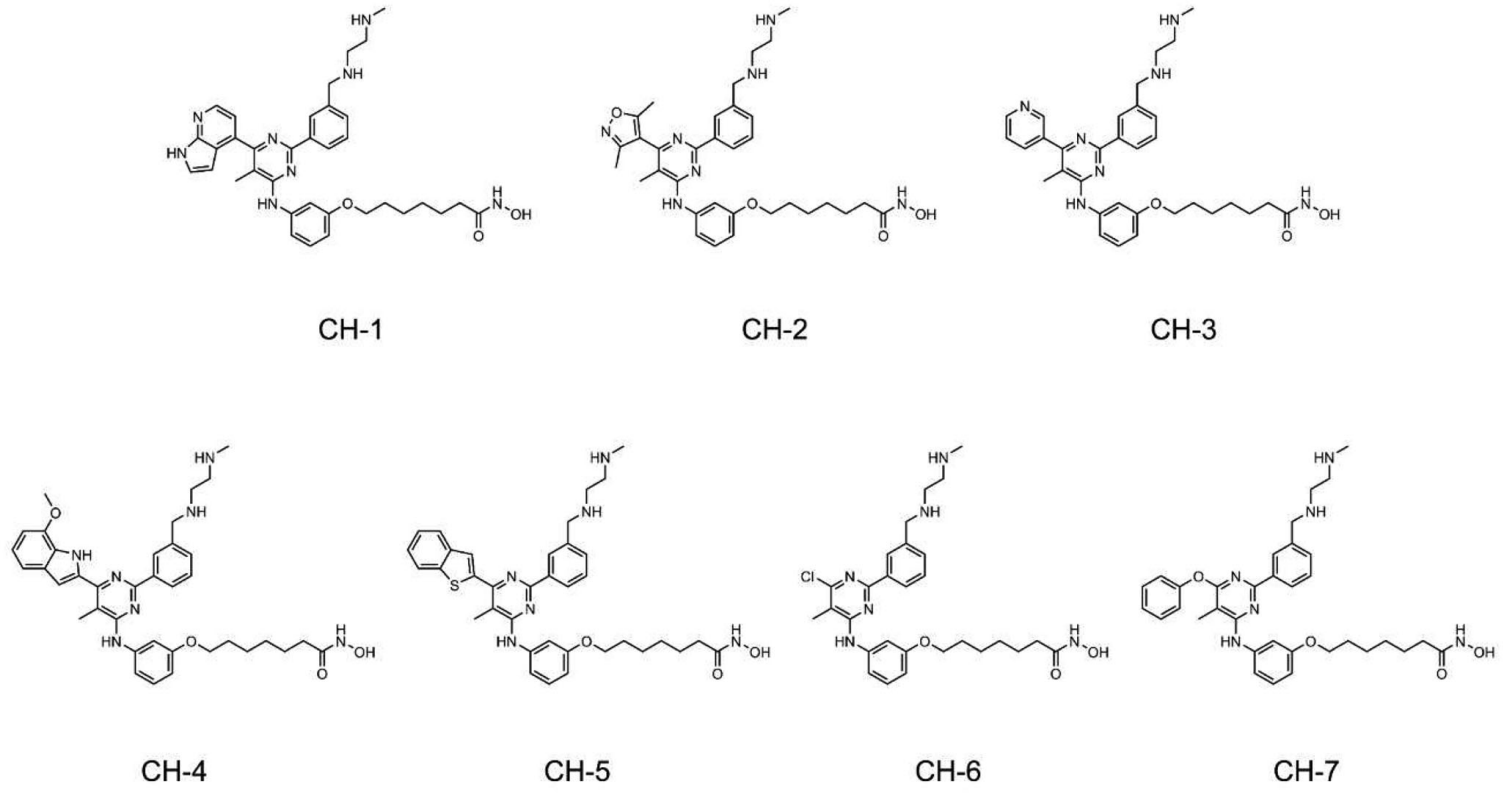
The chemical structures of seven compounds (CHs 1–7).

In this study, the multi-step virtual screening procedure is displayed in Figure 2(B). First, a 2D small molecule database containing 35 000 compounds was constructed for screening. Each compound structure was converted from 2D to 3D by using the Energy Minimisation algorithm. Then, the CARM1 pharmacophore model described above was served as a restriction for screening, and we obtained 139 hit compounds matching all pharmacophore features. Subsequently, the high-resolution crystal structure of CARM1 and HDAC2 (PDB ID: 4LXZ) was retrieved for molecular docking. These 139 compounds were further screened for docking to the active sites of CARM1 and HDAC2, respectively. The docking binding free energy was calculated to predict the binding affinity of each hit to the catalytic active site pocket of the target, with lower values indicating stronger binding affinity. For the CARM1-docking results, we set a rational docking binding energy of ≤11.3 kcal/mol as a cut-off value, and 35 compounds with binding energies lower than −11.3 kcal/mol were selected (Table 1). For the HDAC2-docking results, we obtained 22 compounds with binding energies below −11.7 kcal/mol. Figure 2(C) demonstrated the docking intersection result, we finally obtained seven candidate compounds (termed as CHs 1–7) simultaneously matching two filter criteria (Figure 3). We further investigated interactions between these seven hits and CARM1/HDAC2.

**Table 1.** The docking scores of CHs 1–7.

Compounds	CARM1 binding free energy^a^ (kcal/mol)	HDAC2 binding free energy (kcal/mol)
CH-1	−12.41	−12.37
CH-2	−12.29	−12.12
CH-3	−12.07	−12.25
CH-4	−11.68	−12.09
CH-5	−11.54	−11.86
CH-6	−11.49	−11.94
CH-7	−11.32	−11.71

^a^Binding free energy between the compound and the target (lower binding free energies show stronger binding affinities).

### Validation of pharmacophore model

The specificity and accuracy of the generated structure-based model were further evaluated by the Güner–Henry (GH) score. A database of total 1500 molecules including 1484 inactive and 16 active molecules was used for screening. The results of the statistical parameters were displayed in Table 2. When the GH score is between 0.7 and 1, it indicates that the validated model is excellent. In these validation results, we noticed that the GH score of the CARM1 pharmacophore model is 0.88, which demonstrates a great capacity to distinguish the active molecules from the inactive ones.

**Table 2.** Pharmacophore model validation by GH score method.

Parameter	Pharmacophore model
Total molecules in database (D)	1500
Total number of actives in database (A)	16
Total hits (Ht)	19
Actives hits (Ha)	16
% Yield of actives [(Ha/Ht) × 100]	84%
% Ratio of actives [(Ha/A) × 100]	100%
Enrichment factor (E) [(Ha × D)/(Ht × A)]	79
False negatives (A − Ha)	0
False positives (Ht − Ha)	3
Goodness of hit score (GH)	0.88

### Interaction analysis

The binding pattern of CHs 1–7 to the active regions of CARM1 and HDAC2 proteins was predicted using docking studies. Figure 4 shows the interactions between CHs 1–7 and CARM1. As shown in Figure 4(A,C,E,G,I,K,M), six amino acid residues (Glu266, Glu257, Tyr476, Phe474, Tyr261, Lys470) played an essential role in the binding each compound to CARM1. It is notable that all hit compounds formed stable hydrogen bonds with residues Glu266 and Tyr476, and where CHs 1–5 formed an additional hydrogen bond with Glu257. These interactions enable to stabilise the compound binding to the CARM1 active site and facilitate ligand-protein interactions, allowing for a better fit between the molecule and the protein binding pocket. In particular, CH-1 formed five hydrogen bonds with amino acid residues, with more hydrogen bonds than other hit compounds, corresponding to the highest docking score result. Moreover, the carbon chain and alkyl groups of each hit formed hydrophobic interactions with three residues Phe474, Tyr261, and Tyr476, matching the hydrophobic feature (F4). Figure 4(B,D,F,H,J,L,N) shows the binding surface of CHs 1–7 with CARM1, indicating that the compounds were perfectly matched with the binding pocket of CARM1. After that, the predicted docking binding pattern of CHs 1–7 and HDAC2 is demonstrated in Figure 5. As displayed in Figure 5(A,C,E,G,I,K,M), CHs 1–7 interacted with eight amino acid residues, namely Tyr308, Asp181, His145, His146, Phe210, Asn100, Phe155, and Pro34. The hydroxamic acid group of each compound formed an ionic bond with zinc ion, while formed hydrogen bonds with residues Asp181, Tyr308, and His146. The imine group on the carbon chain formed a hydrogen bond with Asn100. Furthermore, observed from the surface plots of the binding sites (Figure 5(B,D,F,H,J,L,N)), the hydroxamic acid groups of 7 hits entered into the HDAC2 active pocket in the zinc ion binding region. Thus, CHs 1–7 can be perfectly accommodated within the pocket of HDAC2. In general, these docking posture prediction findings reveal that CHs 1–7 can simultaneously interact with key residues of both CARM1 and HDAC2.

Figure 4.X-ray crystal structure and predicted binding modes of CHs 1–7 at the CARM1 active site: (A,B) CH-1; (C,D) CH-2; (E,F) CH-3; (G,H) CH-4; (I,J) CH-5; (K,L) CH-6; (M,N) CH-7. Compounds were indicated as sticks with the atoms coloured as carbon-cyan, nitrogen-blue, oxygen-red, sulphur-yellow and chlorine-green. CARM1 was colour-coded by grey. Hydrogen bonds were indicated by dashed black dotted lines. The surface of CARM1 was shown by mild polar (blue), hydrophobicity (green), and H-bonding (purple) regions.

**Figure:**
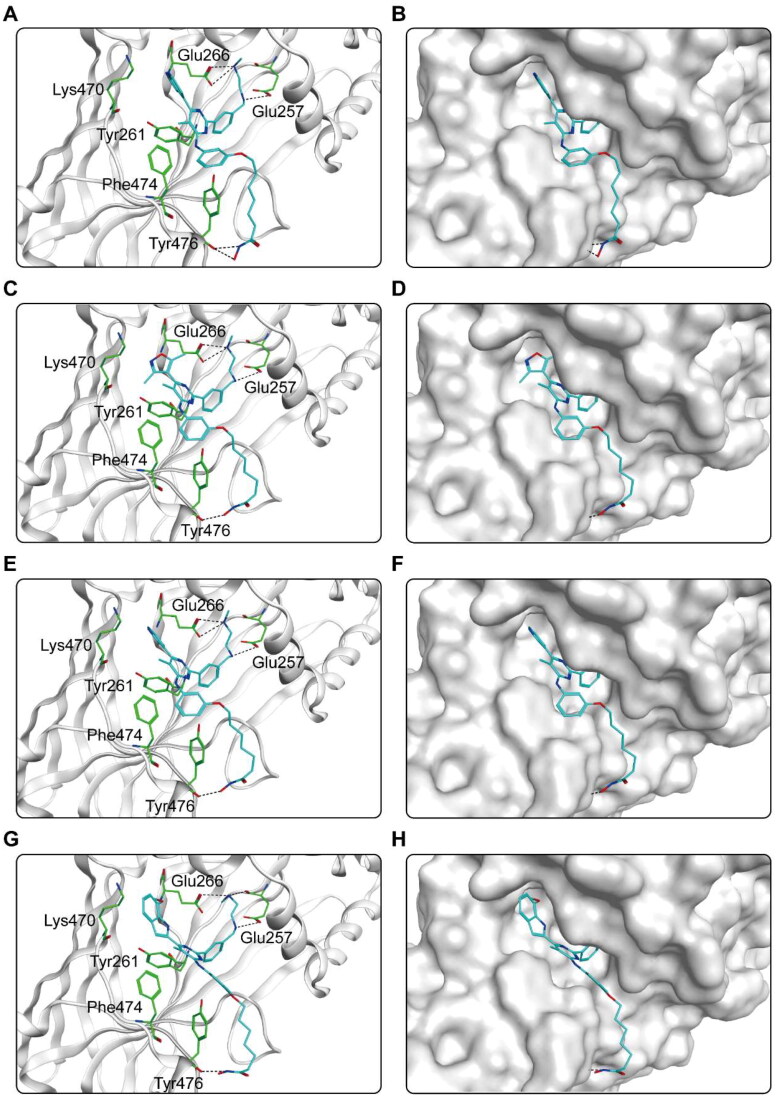

**Figure:**
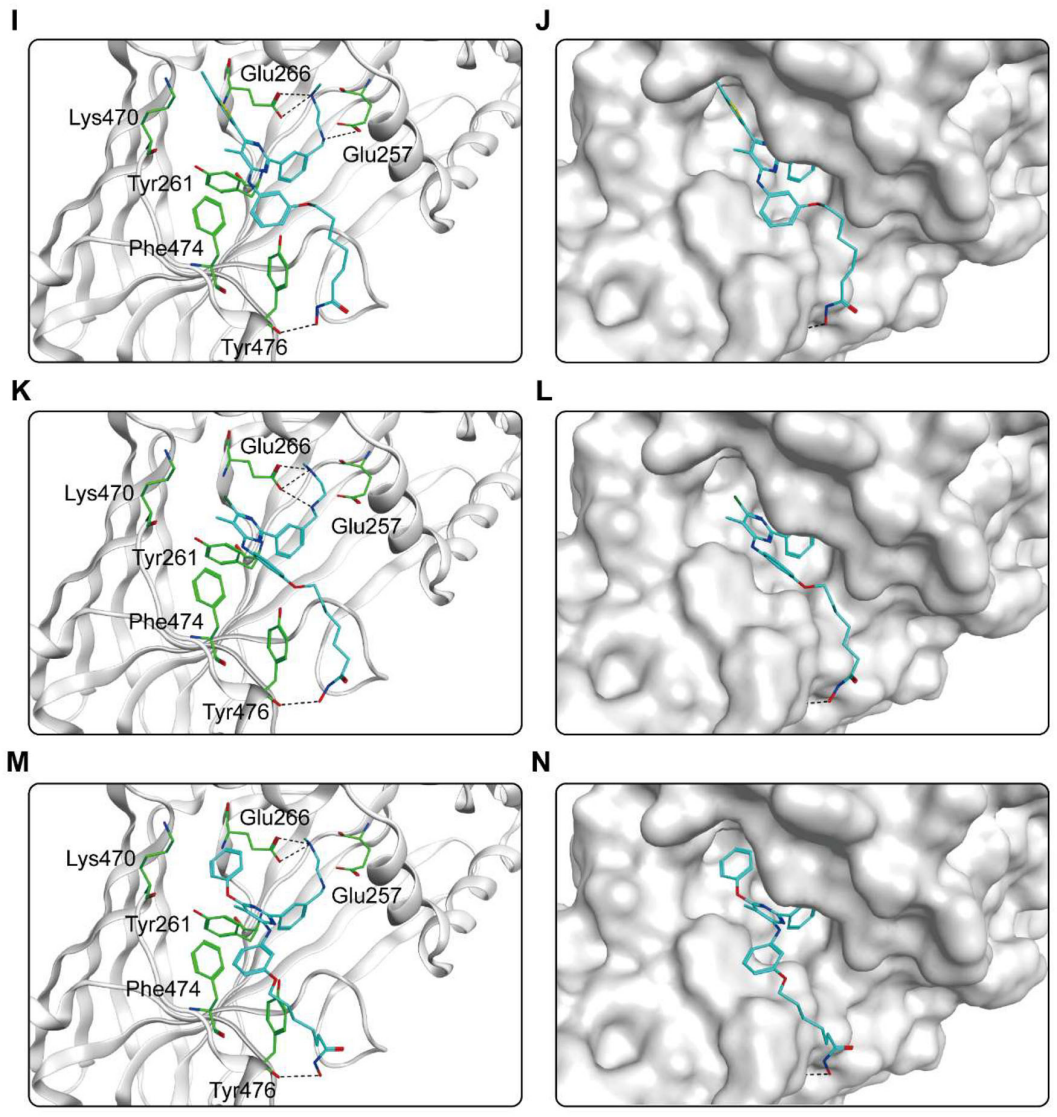

Figure 5.X-ray crystal structure and predicted binding modes of CHs 1–7 at the HDAC2 active site: (A,B) CH-1; (C,D) CH-2; (E,F) CH-3; (G,H) CH-4; (I, J) CH-5; (K,L) CH-6; (M,N) CH-7. Compounds were indicated as sticks with the atoms coloured as carbon-cyan, nitrogen-blue, oxygen-red, sulphur-yellow and chlorine-green. HDAC2 was colour-coded by grey. Hydrogen bonds were indicated by dashed black dotted lines. The surface of HDAC2 was shown by mild polar (blue), hydrophobicity (green), and H-bonding (purple) regions.

**Figure:**
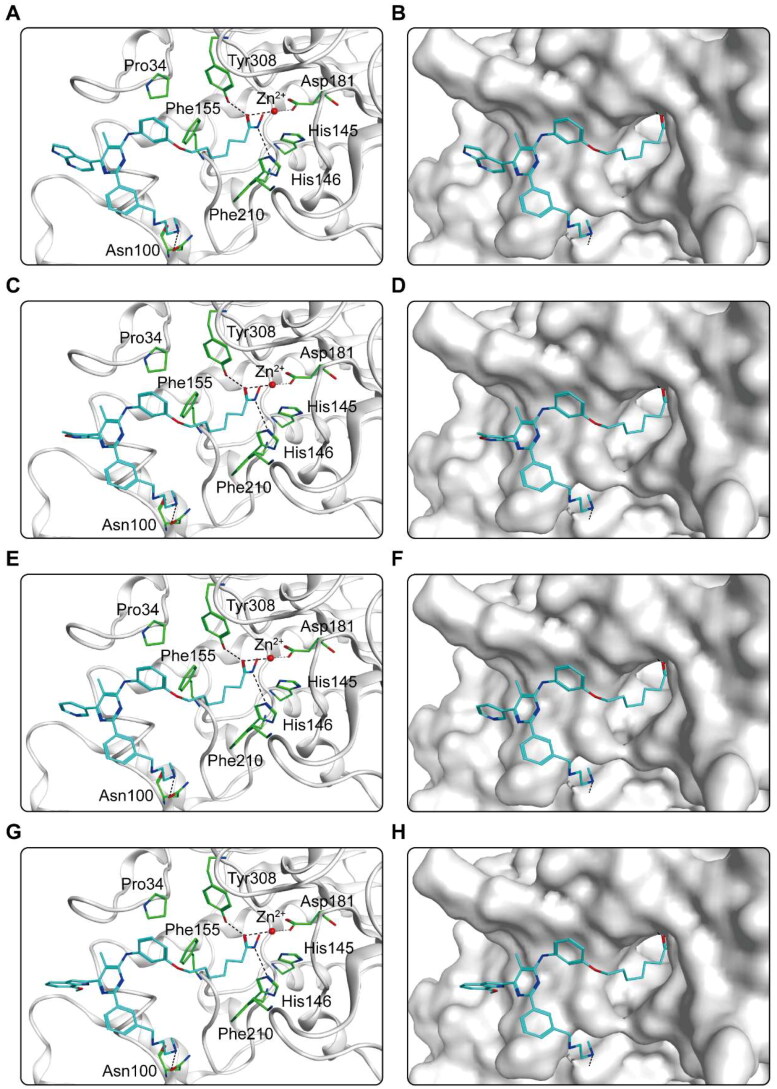

**Figure:**
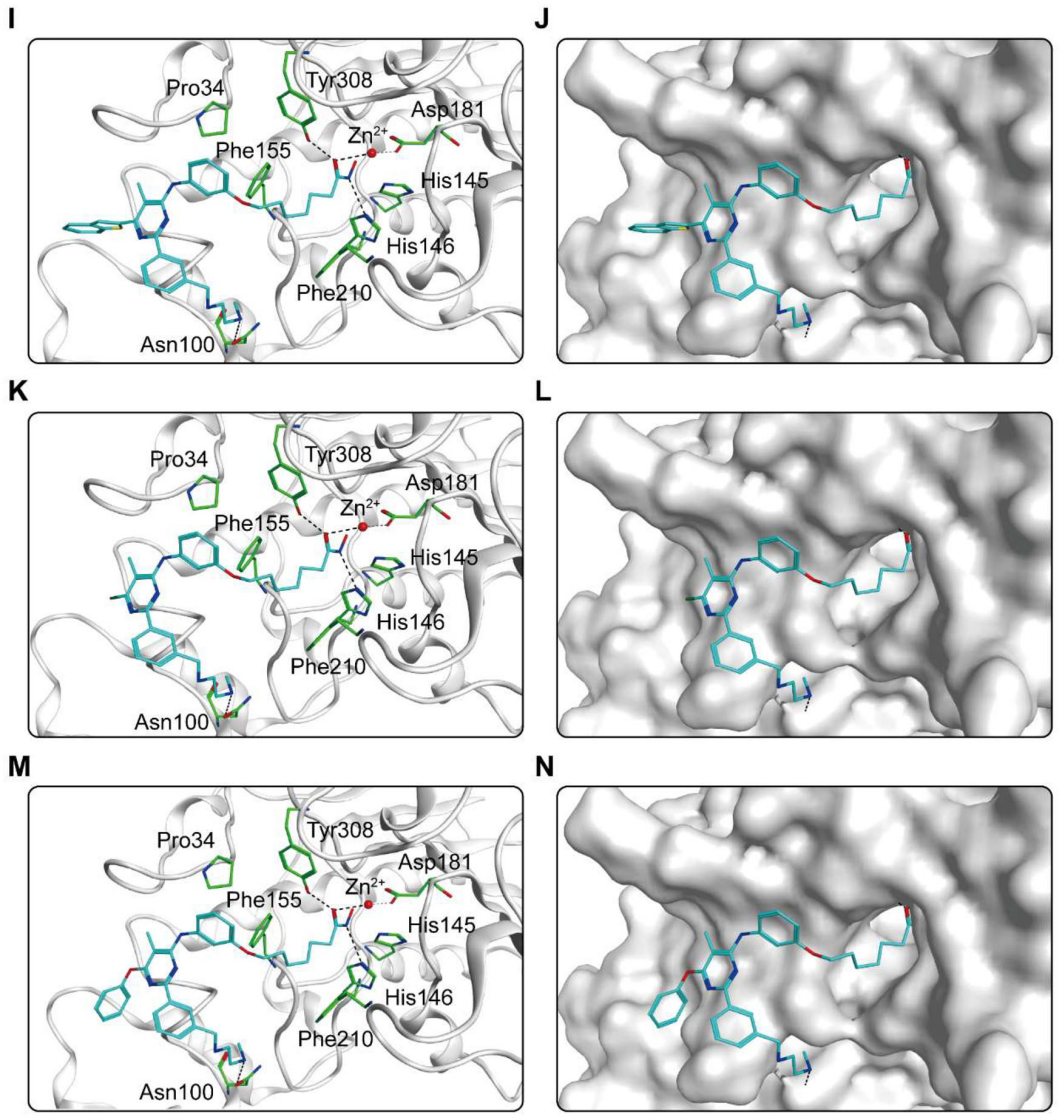

The key protein-ligand interactions of each compound were displayed in Table 3. By analysing these interaction results and referring to the available literature, we further predicted the inhibitory effect of CHs 1–7 on CARM1 and HDAC2. According to the report of CARM1 inhibitors EZM2302, the hydrogen bond with Glu257 and Glu266 are essential interactions to exert inhibitory activity^29^. Notably, CHs 1–5 engaged in hydrogen bonds with Glu257, Glu266, and Tyr476, which had similar hydrogen bond interactions to EZM2302. CH-6 and CH-7 lacked the hydrogen bond with Glu257, and they might be less potent than CHs 1–5. For HDAC2 inhibitor, the hydroxamic acid group established ionic interactions with the zinc ion, which is an essential inhibition mechanism of hydroxamate class HDAC inhibitors^32^. Each compound of CHs 1–7 formed an ionic bond with the zinc ion and formed additional hydrogen bonds with residues located around the zinc, which allowed compounds to lock in the pocket and exert inhibitory activity on HDAC2. Thus, as predicted by the above interaction results, CHs 1–7 can stably bind the active pocket and may have inhibitory effects on both CARM1 and HDAC2.

**Table 3.** The key protein-ligand interactions of CHs 1–7 with CARM1 and HDAC2.

	Interactions with CARM1	Interactions with HDAC2
CH-1	Hydrogen bonds: Glu266, Tyr476, Glu257	Hydrogen bonds: Asp181, Tyr308, His146, Asn100
		An ionic bond with the zinc ion
CH-2	Hydrogen bonds: Glu266, Tyr476, Glu257	Hydrogen bonds: Asp181, Tyr308, His146, Asn100
		An ionic bond with the zinc ion
CH-3	Hydrogen bonds: Glu266, Tyr476, Glu257	Hydrogen bonds: Asp181, Tyr308, His146, Asn100
		An ionic bond with the zinc ion
CH-4	Hydrogen bonds: Glu266, Tyr476, Glu257	Hydrogen bonds: Asp181, Tyr308, His146, Asn100
		An ionic bond with the zinc ion
CH-5	Hydrogen bonds: Glu266, Tyr476, Glu257	Hydrogen bonds: Asp181, Tyr308, His146, Asn100
		An ionic bond with the zinc ion
CH-6	Hydrogen bonds: Glu266, Tyr476	Hydrogen bonds: Asp181, Tyr308, His146, Asn100
		An ionic bond with the zinc ion
CH-7	Hydrogen bonds: Glu266, Tyr476	Hydrogen bonds: Asp181, Tyr308, His146, Asn100
		An ionic bond with the zinc ion

### Inhibition of CARM1 and HDAC2

**Figure 6.**
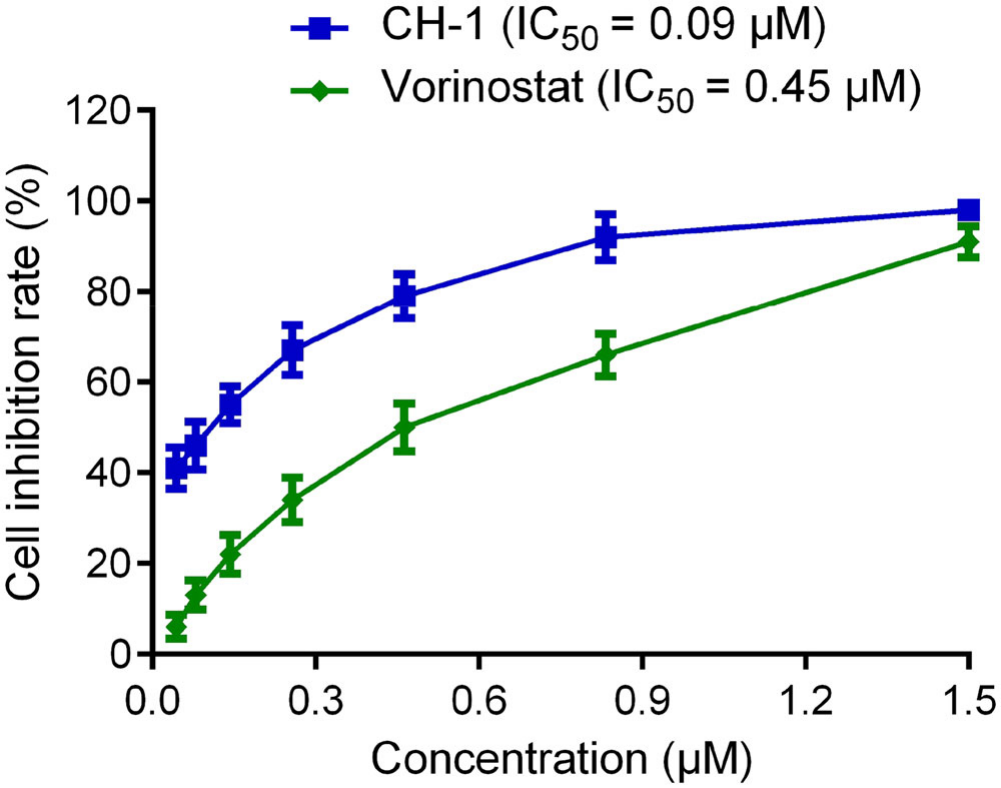
IC_50_ values of CH-1 and vorinostat for DU145 cells. Data are presented as the mean ± SD, *n* = 3.

To further determine the inhibitory effect of CHs 1–7 on CARM1 and HDAC2, the *in vitro* inhibitory assay was performed. EZM2302 and vorinostat were served as positive control drugs. As displayed in Table 4, the 7 hits possessed potent inhibitory activities at the nanomolar level. Among them, CH-1 and CH-2 exhibited higher inhibitory effects, with lower IC_50_ values than the positive control. EZM2302 as a positive control for CARM1 inhibition showed an IC_50_ value of 5.97 ± 0.61 nM, while there was no inhibition of HDAC2. vorinostat showed inhibitory activity to HDAC2 (IC_50_ = 9.53 ± 0.92 nM) and no inhibitory effect on CARM1. Importantly, CH-1 was the most active inhibitor which exhibited excellent and balanced inhibitory effects against both CARM1 (IC_50_ = 3.71 ± 0.11 nM) and HDAC2 (IC_50_ = 4.07 ± 0.25 nM), with an IC_50_ value of about 1.6 times that of EZM2302 and 2.3 times that of vorinostat. In addition, we evaluated the inhibition rate of CHs 1–7 on DU145 cells at a concentration of 1 µM. As the results showed, 6 out of the 7 compounds displayed ≥ 85% inhibition rate. More specifically, the inhibitory effects of CHs 1–4 were higher than that of vorinostat, and the inhibition rate of CH-1 on DU145 cell was up to 97%. As shown in Figure 6, we analysed the inhibition rates of CH-1 and vorinostat at different concentrations and calculated their IC_50_ values on DU145 cells. The inhibitory activity of CH-1 (IC_50_ = 0.09 µM) on DU145 cells is five times stronger than that of vorinostat (IC_50_ = 0.45 µM). Particularly, at low concentrations, the dual inhibitor CH-1 showed a stronger inhibitory effect on DU145 cells than vorinostat. These results indicate that CH-1 has a more significant cell inhibitory ability than vorinostat.

**Table 4.** Inhibitory effects of CHs 1–7, EZM2302 and vorinostat.

Compounds	CARM1	HDAC2	DU145
	(IC_50_, nM)	(IC_50_, nM)	(% inhibition rate, 1 μM)
CH-1	3.71 ± 0.11	4.07 ± 0.25	97%
CH-2	5.38 ± 0.54	8.33 ± 1.05	93%
CH-3	10.55 ± 0.93	5.83 ± 0.41	96%
CH-4	25.54 ± 1.12	9.18 ± 0.17	92%
CH-5	30.14 ± 2.02	17.27 ± 1.96	85%
CH-6	37.37 ± 1.65	12.96 ± 1.25	86%
CH-7	58.48 ± 3.21	24.76 ± 1.53	81%
EZM2302	5.97 ± 0.61	no inhibition	<50%
vorinostat	no inhibition	9.53 ± 0.92	89%

### Molecular dynamics simulation

**Figure 7.**
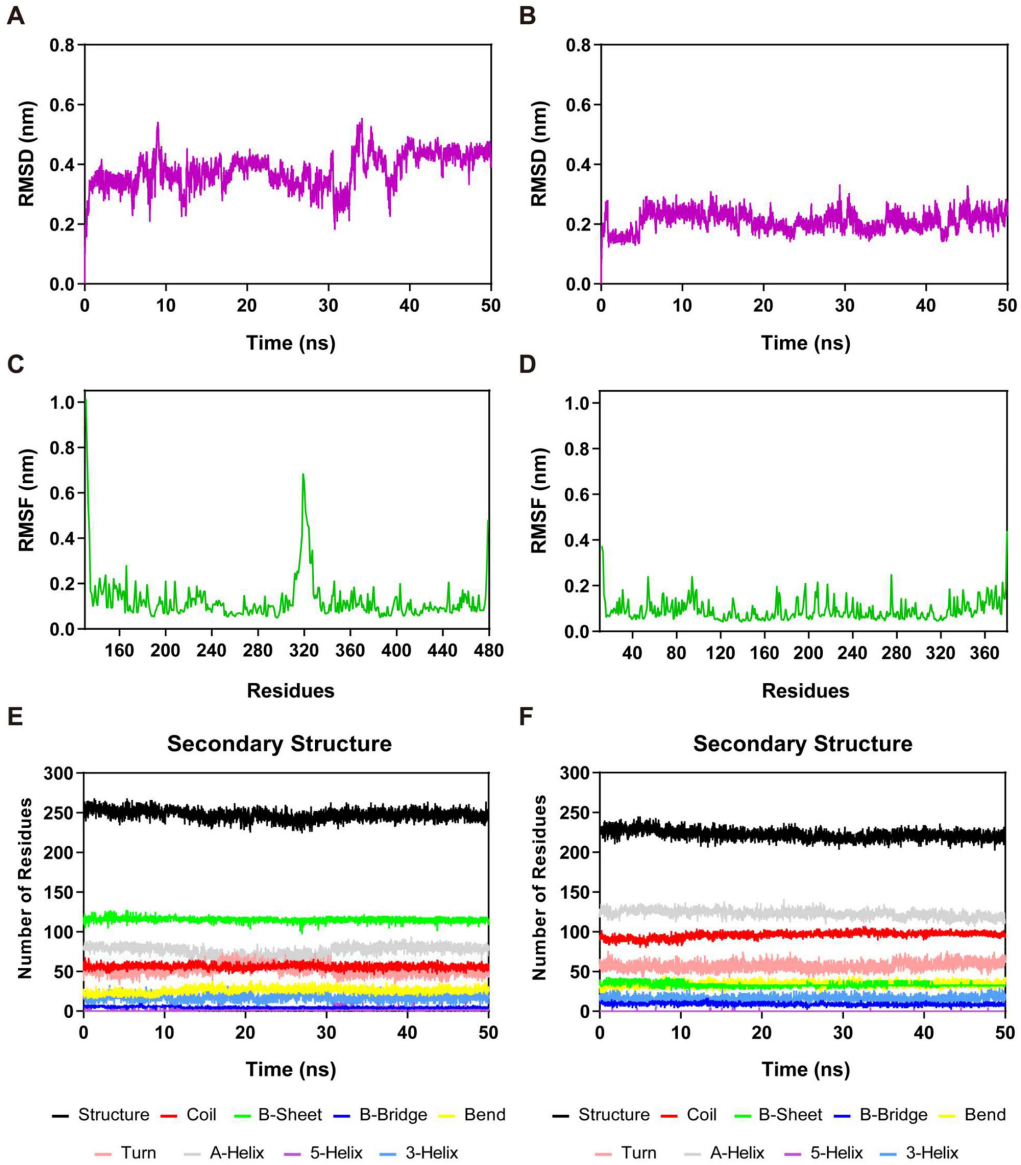
MD simulation of CH-1 in complex with CARM1 and HDAC2. (A) RMSD of CH-1 in CARM1-CH-1 complex. (B) RMSD of CH-1 in HDAC2-CH-1 complex. (C) RMSF of Cα atoms of CARM1 residues in CARM1-CH-1 complex. (D) RMSF of Cα atoms of HDAC2 residues in HDAC2-CH-1 complex. (E,F) The secondary structures analysis of CH-1 in complex with CARM1 and HDAC2, respectively.

Therefore, to verify whether CH-1 can be stably bound within the cavity of CARM1 and HDAC2, we conducted 50 ns MD simulations. The results of the MD simulation were presented by monitoring root mean square deviation (RMSD), root mean square fluctuation (RMSF), and secondary structural changes. Figure 7(A,B) depicted the RMSD of the Cα atoms of CH-1 in complex with CARM1 and HDAC2 proteins. RMSD is used to analyse the stability and reliability of the system. A lower RMSD value and constant fluctuation tend to imply higher binding stability. In the CARM1 complex, we observed that the RMSD reached equilibrium after 40 ns, and then stabilised at about 0.45 nm for the remaining time. In the HDAC2 complex, the RMSD reached equilibrium after 5 ns, followed by a slight increase, and finally stabilised at roughly 0.23 nm. Thus, the average RMSD values of CH-1 in these two complex systems are both below 0.4 nm and gradually converge to equilibrium. We considered that CH-1 was quite stable in the complexes during the 50 ns simulation. RMSF values reveal structural fluctuations and flexibility in the protein, and a lower RMSF value indicates better protein stability. Figure 7(C,D) revealed the RMSF of the Cα atoms of residues in the CARM1 complex and HDAC2 complex, respectively. In the CARM1 complex, residues Glu266, Glu257, Tyr476, Phe474, Tyr261, and Lys470, which formed key interactions with CH-1, exhibited small RMSF fluctuations in the < 0.2 nm range. In addition, the residue 315 to 321 within the loop region displayed a higher flexibility, but these residues are located far from the binding site. In the HDAC2 complex, the key residues (Tyr308, Asp181, His145, His146, Phe210, Asn100, Phe155, and Pro34) binding to CH-1 showed limited fluctuations below 0.2 nm. In addition, both complexes showed high fluctuations in the C-/N -ends, which might be related to their few interactions. As shown in Figure 7(E,F), we further simulated the secondary structures of these two protein complexes, including Coil, B-Sheet, B-Bridge, Bend, Turn, A-Helix, 3-Helix, and 5-Helix. These secondary structures were in a stable state of fluctuation within 50 ns. Overall, MD findings reveal that CH-1 could be tightly and simultaneously attached to the active pockets of CARM1 and HDAC2.

### Cell growth inhibition

As previously stated, compound CH-1 exhibited the strongest inhibitory effect. Thus, CH-1 was further evaluated for anti-proliferative effect against prostate cancer-associated cells. DU145, RM-1, LAPC4, 22RV1, and PC-3 cells were treated with CH-1, and their half maximum inhibitory concentration (IC_50_) values were determined by MTT assay. We detected the cell proliferation inhibiting ability of CH-1 on these five cell lines. As shown in Table 5, CH-1 displayed significant growth inhibitory activity against tumour cells (IC_50_ < 1 µM). In particular, the strongest inhibitory effect was observed on the DU145 cell with 0.09 µM. In brief, the results of the cell growth inhibition assay indicate that CH-1 has a strong ability to reduce human prostate cancer cell proliferation.

**Table 5.** Cell growth inhibition of CH-1.

Assay	IC_50_ (μM)
DU145 cell proliferation	0.09
RM-1 cell proliferation	0.71
LAPC4 cell proliferation	0.68
22RV1 cell proliferation	0.95
PC-3 cell proliferation	0.43

### In vivo tumour growth inhibition

**Figure 8.**
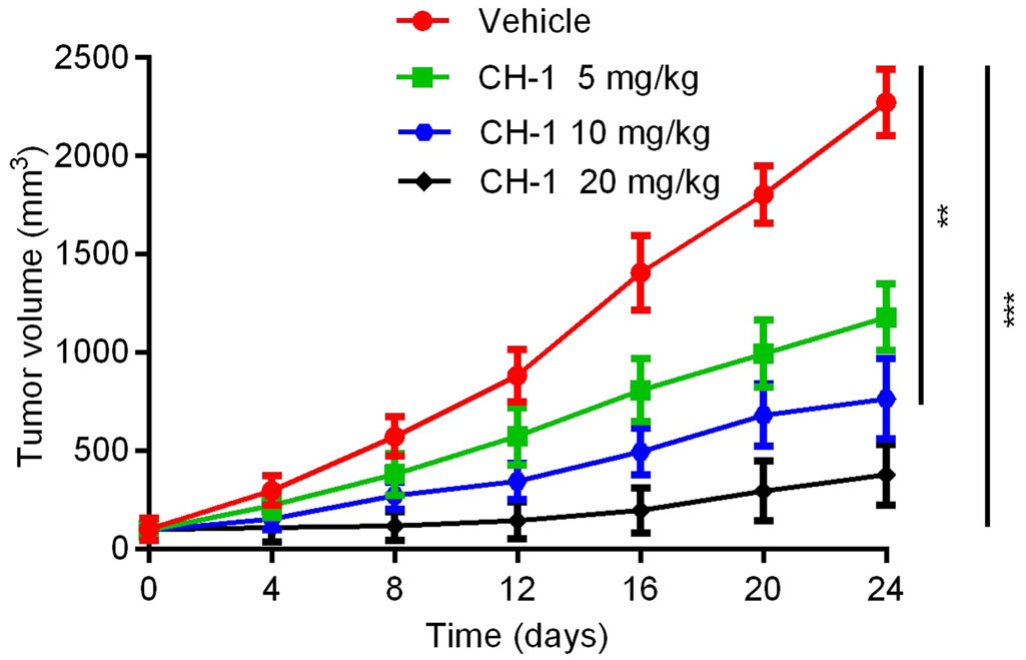
The tumour volume changed with time at different CH-1 concentrations. Data are presented as the mean ± SD, *n* = 3. ***p < 0.001 and **p < 0.01 mean a significant difference *versus* the vehicle group.

A xenograft tumour model derived from human prostate cancer cell was established in nude mice to further validate the antitumour activity of CH-1 *in vivo*. The tumour-bearing mice were divided into 4 groups: vehicle, CH-1 (5 mg/kg), CH-1 (10 mg/kg), and CH-1 (20 mg/kg). Apparently, the tumour volume in the 5 mg/kg, 10 mg/kg, and 20 mg/kg dose groups treated by CH-1 was significantly reduced compared with the control group (Figure 8), indicating that CH-1 has a strong anticancer effect *in vivo*. Furthermore, mice treated with CH-1 20 mg/kg dose group had the most excellent inhibitory effect against tumour volume growth in mice, indicating that CH-1 inhibits tumour growth in a dose-dependent manner. Therefore, experimental data suggest that compound CH-1 has great *in vivo* antitumour activity.

## Discussion

PCa is a terrifyingly heterogeneous disease, and many risk factors increase the possibility of the disease occurrence. Over the past five years, the incidence of prostate cancer has increased by approximately 3% annually. Currently, single-target agents are limited in treating heterogeneous and complex diseases. There is an urgent need to discover novel and highly efficient drugs. In summary, we rationally designed CHs 1–7 as potent and selective CARM1/HDAC2 inhibitors via a virtual screening approach. By analysing interactions between CHs 1–7 and target proteins, we found that CHs 1–7 formed hydrogen bonding interactions and hydrophobic interactions with key residues, blocking the whole CARM1 binding surface. These facilitated the compound to occupy a cavity in the CARM1 active site. Meanwhile, CHs 1–7 bound to key residues of HDAC2, ensuring they could be completely accommodated by the pockets of both CARM1 and HDAC2. Further inhibition assay demonstrated that all compounds showed strong *in vitro* affinity for both CARM1 and HDAC2 at the nanomolar level. CH-1 proved to be the most active compound, with an IC_50_ value of about 1.6 times that of CARM1 positive control and 2.3 times that of HDAC2 positive control. MD simulations reflected the well-bound affinity and stability of CH-1 in CARM1 and HDAC2 complexes. Importantly, CH-1 displayed effective anti-proliferative ability in multiple prostate-related tumour cell lines, and the inhibition rate of CH-1 on DU145 cells was up to 97%. *In vivo* results showed that CH-1 exerted significant antitumour activity in DU145 xenograft tumour mouse models in a dose-dependent manner.

In this virtual screening methodology, we set rational docking scoring thresholds of CARM1 and HDAC2 docking respectively. The docking score of candidate compounds should be better than both thresholds. Although these compounds possess similar structures, they showed different binding free energies for the CARM1 and HDAC2 docking. Subsequently, we compared the CARM1 binding free energy and HDAC2 binding free energy respectively for each compound and ranked the scores, and analysed them in combination with the inhibitory effects on CARM1 and HDAC2. Most notably, we found that these experimental data are quite consistent with the docking score results. We further analysed the relationship between cytotoxicity assay results and docking scoring results. Obviously, there was also reasonable concordance between the two sets of results, corroborating the rationality of our computational docking predictions. Among all compounds, CH-1 was selected with the lowest binding free energy. CH-1 equally exhibited the most excellent inhibitory effects against both CARM1 and HDAC2, corresponding to the docking score result. The cytotoxicity of CH-1 to DU145 cells was also higher than that of other compounds. Thus, these data further confirm that our virtual screening methodology is robust and reliable. Collectively, we successfully identified a novel CARM1/HDAC2 dual-targeting inhibitor and it may play an essential value in the treatment of malignant tumour.
